# A Digital Platform to Support HIV Case Management for Youth and Young Adults: Mixed Methods Feasibility Study

**DOI:** 10.2196/39357

**Published:** 2022-11-21

**Authors:** Connie Fee, Julia Fuller, Carly E Guss, Elizabeth R Woods, Ellen R Cooper, Urmi Bhaumik, Dionne Graham, Sandra K Burchett, Olivia Dumont, Emily B Martey, Maria Narvaez, Jessica E Haberer, Dallas Swendeman, Shelagh A Mulvaney, Vikram S Kumar, Jonathan L Jackson, Y Xian Ho

**Affiliations:** 1 University of San Francisco San Francisco, CA United States; 2 Dimagi, Inc Cambridge, MA United States; 3 Division of Adolescent/Young Adult Medicine Boston Children's Hospital Boston, MA United States; 4 Harvard Medical School Boston, MA United States; 5 Boston University School of Medicine Boston, MA United States; 6 Division of Pediatric Infectious Diseases Boston Medical Center Boston, MA United States; 7 Program for Patient Safety & Quality Boston Children’s Hospital Boston, MA United States; 8 Division of Infectious Diseases Boston Children's Hospital Boston, MA United States; 9 Massachusetts General Hospital Boston, MA United States; 10 Department of Psychiatry and Biobehavioral Sciences David Geffen School of Medicine University of California Los Angeles, CA United States; 11 School of Nursing Vanderbilt University Nashville, TN United States

**Keywords:** HIV, case management, youth, young adult, mobile health, mHealth, digital health, mobile phone

## Abstract

**Background:**

Advances in medical treatments in recent years have contributed to an overall decline in HIV-related opportunistic infections and deaths in youth; however, mortality and morbidity rates in perinatally and nonperinatally infected adolescents and young adults (AYA) living with HIV remain relatively high today.

**Objective:**

The goal of this project was to assess the use, utility, and cost-effectiveness of *PlusCare,* a digital app for HIV case management in AYA living with HIV. The app supports routine case management tasks, such as scheduling follow-up visits, sharing documents for review and signature, laboratory test results, and between-visit communications (eg, encouraging messages).

**Methods:**

We conducted a single-group mixed methods pre-post study with HIV case management programs in 2 large urban hospitals in the Boston metro area. Case management staff (case managers [CMs], N=20) and AYA living with HIV participants (N=45) took part in the study with access to PlusCare for up to 15 and 12 months, respectively.

**Results:**

The CMs and AYA living with HIV reported mean System Usability Scale scores of 51 (SD 7.9) and 63 (SD 10.6), respectively. Although marginally significant, total charges billed at 1 of the 2 sites compared with the 12 months before app use (including emergency, inpatient, and outpatient charges) decreased by 41% (*P*=.046). We also observed slight increases in AYA living with HIV self-reported self-efficacy in chronic disease management and quality of life (Health-Related Quality of Life-4) from baseline to the 12-month follow-up (*P=*.02 and *P*=.03, respectively) and increased self-efficacy from the 6- to 12-month follow-up (*P*=.02). There was no significant change in HIV viral suppression, appointment adherence, or medication adherence in this small-sample pilot study.

**Conclusions:**

Although perceived usability was low, qualitative feedback from CMs and use patterns suggested that direct messaging and timely, remote, and secure sharing of laboratory results and documents (including electronic signatures) between CMs and AYA living with HIV can be particularly useful and have potential value in supporting care coordination and promoting patient self-efficacy and quality of life.

**Trial Registration:**

ClinicalTrials.gov NCT03758066; https://clinicaltrials.gov/ct2/show/NCT03758066

## Introduction

### Background

Adolescents and young adults (AYA) are estimated to account for more than 1 in 5 new HIV diagnoses in the United States [1]. Advances in medical treatments and combination antiretroviral therapy have contributed to an overall decline in HIV-related opportunistic infections and deaths among youth in the United States and other high-income countries [2-5]. However, rates of mortality and morbidity in perinatally and behaviorally infected AYA living with HIV remain high in the United States, partly owing to poor HIV control [6]. By the end of 2019, it was estimated that only 35% of persons aged 13 to 24 years with HIV were virally suppressed and only 33% were retained in care, the lowest percentages for any age group [7]. AYA living with HIV require developmentally appropriate care and case management to achieve retention in care and viral suppression, as well as other developmental and health outcomes, as they become more responsible for their own care and adherence at the same time that they enter the turbulence of adolescent and young adult transitions [8,9].

Case management programs can help AYA living with HIV work toward achieving primary HIV outcomes, such as viral suppression, by providing support for fundamental HIV care needs, in addition to non–HIV-related health and developmental outcomes that may co-occur with or undermine HIV outcomes [10]. The use of case management has been associated with decreased unmet needs for supportive care of AYA living with HIV, such as health insurance, emotional counseling, and higher medication adherence in patients receiving HIV treatment [11]. Case managers (CMs) and other clinical care staff who serve as part of the case management team, such as physicians, nurse practitioners, nurses, and social workers (hereafter collectively referred to as CMs), play a vital role in the front line of care throughout the HIV care continuum, often starting with reporting a positive test result to a patient and then connecting youth to resources that can assist in fulfilling basic needs including insurance, medical care, housing, and transportation, as well as mental health and general emotional support [12-14]. Engagement and retention in care become critical as AYA living with HIV age and transition into adult care, and studies suggest that case management can contribute to the retention of AYA living with HIV in primary care, particularly in high-risk populations [15-19], by providing linkage to care most needed by hard-to-reach populations [14,20-25]. Logistical barriers, such as arrangements for transportation to the hospital or clinic, can prevent AYA living with HIV from receiving the care that they need. A case management team can support the fundamental aspects of AYA living with HIV care but is limited by constraints such as infrequent clinic visits and unreliable means of communication with patients.

Mobile health (mHealth) technology provides an opportunity to overcome these limitations and facilitates case management for AYA living with HIV. Pew research reported an estimated 95% of adolescents in the United States in 2018 owning or having access to a smartphone, and numbers are likely to be higher today [26]. The pervasiveness of mobile technology in the youth today, combined with its unique capabilities to securely handle data, presents a distinctive opportunity to support AYA living with HIV [27]. Although there have been many advances in mHealth to support persons living with HIV/AIDS, few tools have been developed to directly support case management through a shared platform between the clinical care team and AYA living with HIV, a capability that is desired and has the potential to streamline care [28]. Unlike adults living with HIV, AYA living with HIV likely require a more complex, individualized, youth-friendly approach provided by case management programs [29]. We designed and previously found high perceived usability of the prototype of a digital app system, *PlusCare*, to support HIV case management for AYA living with HIV, demonstrating its acceptability and feasibility [30]. Using a user-centered design approach, we developed a mobile and web app that can directly connect AYA living with HIV and members of their case management team by streamlining communication and facilitating comprehensive support to help overcome logistical barriers to care [31].

### Objectives

The objective of this study was to assess the user experience of the PlusCare system for AYA living with HIV case management, cost-effectiveness, and potential effects on physical and psychosocial health.

## Methods

A mixed methods, nonrandomized, single-group intervention study design (ClinicalTrials.gov NCT03758066) was used.

### Study Settings

The study was conducted with 3 HIV/AIDS programs: 2 based at Boston Children’s Hospital (BCH), and 1 at Boston Medical Center (BMC). BCH’s programs are housed in both adolescent and young adult medicine and infectious diseases and BMC’s program belongs to the infectious diseases division. All 3 programs shared a similar focus on HIV counseling, prevention, screening, and linkage to care, retention, and adherence. They used a model of multidisciplinary team-managed care in which each AYA living with HIV is assigned to a designated team consisting of, at minimum, a CM, a medical provider, and a nurse. Program staff at both sites communicated similarly with patients, relying primarily on phone calls and in-person visits to connect with patients; for example, to share and discuss laboratory results and collect signatures on documents. At BCH, CMs also communicated with patients via the patient portal, and at BMC, SMS text messaging was occasionally used; both modalities were used for less confidential communication. Paper binders and electronic health record systems were used at both sites to document interactions. BCH and BMC are private, not-for-profit academic hospitals. BMC is the largest safety-net hospital in the region; that is, it serves a racially and ethnically diverse patient population with a large proportion of patients either uncovered or covered by Medicaid in commitment to its mission statement to provide health care to all patients, regardless of their ability to pay.

### Recruitment and Eligibility

CMs at each site actively involved in the care of AYA living with HIV were identified and referred to the study site research assistant (RA) to confirm eligibility, consent, and enrollment in the study. Patients were eligible to participate if they were aged 13 to 25 years, living with HIV, had reliable access to a smartphone for 12 months, and were at the study site for at least 12 months before enrollment. A waiver of parental consent for participants aged 13 to 17 years was approved by the institutional review board (IRB) to protect participant confidentiality. All participants were recruited and enrolled between January 2019 and June 2019.

### Baseline and App Training

CMs from each of the 3 programs participated in a baseline session that took place in January 2019. These sessions were led by study site RAs at each site who had in-depth knowledge and experience with the app. Written informed consent was obtained from each CM at the beginning of each session. A baseline survey was conducted to collect basic demographic data and information on professional roles and experience (eg, job title, years of CM experience, and number of AYA living with HIV managed). In this session, CMs received training on how to use the PlusCare mobile app and register AYA living with HIV participants.

RAs consented to the participation of eligible AYA living with HIV and then completed a baseline survey which included items to collect demographic information and assess technology use, familiarity, and health outcome measures. CMs were responsible for registering consented and enrolled patients in the PlusCare system and setting up the patient’s care team contacts. Only the web app was tested with the patients at enrollment to ensure equivalent access agnostic to operating system, although PlusCare could theoretically be accessed as a mobile app via Android devices. A shortcut to the web app was created on the participants’ devices for easy access. Baseline training was considered complete after the PlusCare shortcut was installed and the participant successfully demonstrated that they could (1) access the app from their smartphone and (2) receive incoming SMS text messages from the CM.

### Intervention

Study tablets with PlusCare preinstalled as a native app were provided to the case management programs at each site for use by CMs. CMs could access PlusCare via the app on the study tablets or the web app from any computer for up to 15 months from the start date of the study intervention to account for rolling patient enrollment. Patients had access to the PlusCare web app for 12 months after enrollment in the study and continued to receive usual care from their respective case management teams during that time, including communication with patients about upcoming visits, discussion about laboratory results, updating insurance information, renewing medications, and assistance with social determinants of health needs, such as food, housing, and transportation.

To assess the naturalistic use of the app, user-suggested modifications during the intervention period were restricted to those determined to be barriers to use. As such, only one modification was implemented and introduced in early August 2019, after all participants were enrolled, to allow CMs and patients to send asynchronous messages to one another within the app. Before this modification, only CMs could send messages to patients. However, patients were unable to send messages in response to CMs.

### Outcomes and Measures

In this study, we evaluated (1) use, usability, and acceptability and (2) health and clinical outcomes (CD4 counts, viral load, clinic visits, medication adherence, self-efficacy in chronic disease management, quality of life, and cost savings).

#### Use, Usability, and Acceptability

The 10-item Likert-scale positive System Usability Scale (SUS) [32] was administered via a survey in the PlusCare app to assess CMs’ and patients’ perceived usability of and user satisfaction with the app at the end of the study. The SUS has been validated for use with mobile and web apps and scores range from 0 to 100, with higher scores indicating higher perceived usability [33]. A score >68 is considered above the industry average [34].

Data on which features of the PlusCare app were accessed (ie, forms that users submitted) were also collected passively and deidentified weekly use logs were analyzed separately for CMs and patients to explore use trends over the 15- and 12- month period, respectively.

RAs also conducted brief, structured *check-ins* following an interview guide with CMs every 6 weeks via phone or in person to address and log any issues with their own user experience and their patients. At the end of the study period, the RAs at each site also conducted brief structured one-on-one interviews with CMs at their respective sites to provide qualitative feedback on their experiences using the system with their patients. Qualitative notes were collected for analyses (see Multimedia Appendices 1 and 2 for sample guides used for check-ins and interviews).

#### Health and Clinical Outcomes

Viral load, CD4 counts, and scheduled clinic visits were compared between 1 year before enrollment (pre-enrollment) and 1 year during intervention (postenrollment). Values for patient laboratory results and medical visit frequency were obtained from electronic health records.

Once a month, patients completed a medication adherence assessment, where they rated their adherence to their prescribed medication for the past month on a 6-point Likert scale ranging from 1 (“very poor”) to 6 (“excellent”) [35,36]. Patients received monthly SMS text messages alerting them to report adherence via survey forms created in the PlusCare app.

Responses to self-efficacy and health-related quality of life assessments, the self-efficacy for managing chronic diseases 6-item scale [37] and modified Centers for Disease Control and Prevention Health-Related Quality of Life-4 (HRQOL-4) healthy days core measures [38], respectively, were similarly collected via survey forms in the PlusCare app at baseline, 6 months, and 12 months. Self-efficacy scores were calculated by averaging patients’ self-reported confidence in keeping various symptoms from interfering with their everyday life and their confidence in engaging in activities that reduced the impact of their illness and the need to visit a clinician.

Outpatient, emergency, and inpatient per-patient charges were extracted from hospital billing data as deidentified data sets for analyses.

### Data Analysis

#### Overview

Our analytical sample included 20 CMs and 45 patients enrolled in the study. The sample size of CMs was a convenience sample determined based on feasibility and the pool of potentially eligible case management staff at the 2 sites. The effect size was calculated using Cohen *d*=0.35 and the ability to detect a 38% increase in viral load, independent of sample size [39]. All statistical tests were based on 2-tailed alternatives at a significance level of .05 and analyses were performed using open-source R statistical software (R Foundation for Statistical Computing) [40] and SAS software (version 9.4; SAS Inc) [41].

To assess any differences between sites, baseline demographic characteristics for CMs and patients were compared between sites using 2-sample, 2-tailed *t* tests for averages (ie, age and savviness with technology) and chi-square tests or Fisher exact tests for categorical variables (ie, ethnicity and race, sex, education, smartphone ownership, hours on phone, and health and HIV apps). Missing responses were excluded from the significance tests.

#### Use, Usability, and Acceptability

Descriptive statistics (mean, median, SD, and frequency) were used to summarize SUS scores collected at the end of the study and use patterns in data collected passively over the intervention period. Qualitative notes from check-ins and end-of-study interviews with CMs were coded and analyzed by 2 coauthors (CF and YXH) using directed content analysis with predefined categories of (1) facilitators, (2) barriers, and (3) recommendations for system implementation. Recurring themes in the responses were iteratively coded, reviewed, and refined [42].

#### Health and Clinical Outcomes

We used simple linear regression to test whether self-reported medication adherence improved over the course of the intervention. A 2-tailed nonparametric 1-sample paired sign test was used to determine whether to reject or fail to reject the null hypothesis that the mean difference of self-efficacy for managing chronic diseases 6-item scale and Centers for Disease Control and Prevention HRQOL-4 scores between any of the 2 of 3 time points was 0 (baseline vs 6-month, 6-month vs 12-month, and baseline vs 12-month).

Generalized linear mixed modeling was used to determine changes in HIV viral load and CD4 levels in the preintervention and postintervention periods. Owing to the skewed distribution of the outcome, we log transformed the values before modeling. We included random intercepts for each participant to control for the correlation between repeated measurements within the patient.

Cost-effectiveness analyses were performed following an approach used in other community programs [43-46]. We used data on HIV medical visit frequency, emergency department visits, and hospitalizations for the analyses. We based our analyses on potential cost savings resulting from a reduction in hospital visit frequency between the preintervention period (12 months before baseline) and the intervention period from baseline to the 12-month follow-up. We computed preliminary cost-effectiveness of the PlusCare intervention by dividing the cost savings attained by the program cost of the case management program using the net present value (*net present value = present value of cost savings − present value of program costs*) and return on investment (*return on investment = present value of cost savings / present value of program costs*). The cost savings estimate was adjusted for variations in age, sex, and race and ethnicity using a multivariate regression model to reduce treatment costs.

### Ethics Approval

IRB approval for all study activities was obtained from and overseen by the Western Institutional Review Board (WCG IRB 120190387) and the IRB at each study site (IRB-P00029517 and H-38254).

## Results

### mHealth Study Tool

#### Overview

**Figure 1:**
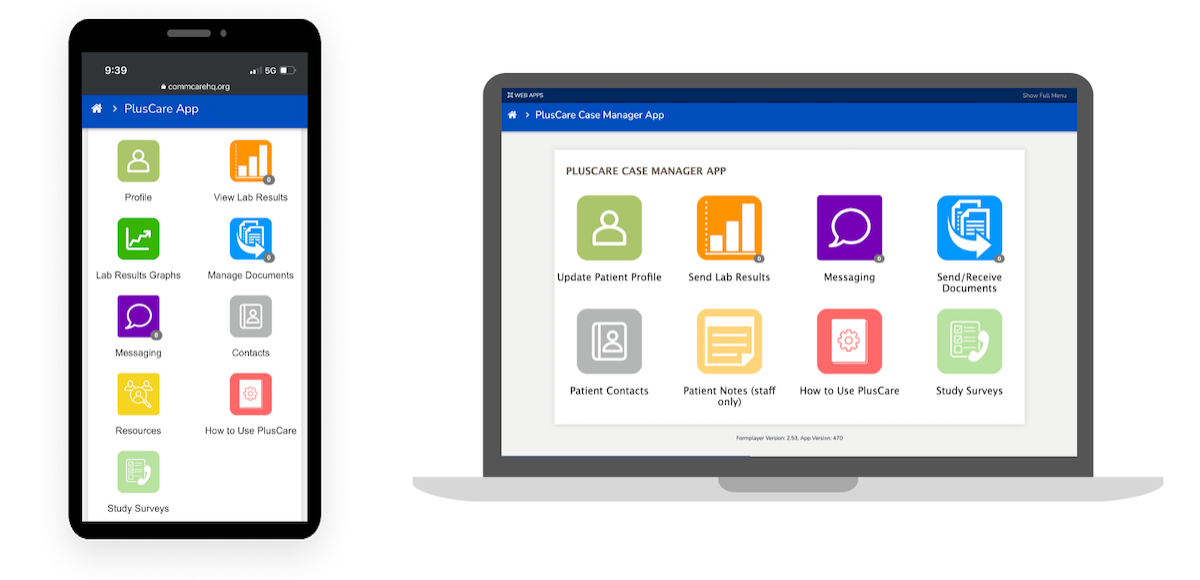
PlusCare dashboard for patient (left) as viewed on a smartphone and case manager as viewed in a web application on a laptop computer (right).

PlusCare was designed with 2 separate interfaces connected on a shared platform—one for CMs and one for the AYA living with HIV. The former can access the app via a native app installed on a study tablet or a web app through a computer browser. Patients could access the web app through a shortcut link on their smartphones. Figure 1 shows screenshots of the menu page for the 2 different users: CMs and patients. The app can support critical aspects of AYA living with HIV case management, and the following functionalities were validated through usability testing (corresponding menu items on the patient and CM interfaces shown in Figure 1 are indicated in parentheses):(1) enter, share, and view laboratory results (View Lab Results, Lab Results Graphs, and Send Lab Results); (2) send, receive, and sign documents via an external document sharing app, Docusign [31] (Manage Documents and Send and Receive Documents); (3) create and update contacts (Contacts and Patient Contacts); (4) schedule, send, and receive SMS text message notifications of app activity, for example, laboratory results, appointment reminders, and encouraging messages (Messaging); and (5) view and go to links to community resources (Resources). In addition, CMs could update patient information registered in the system (Update Patient Profile) and create and share notes between staff (Patient Notes). Both users could access user guides in the system at any time (How to Use PlusCare).

#### Participant Demographics

A total of 20 CMs were enrolled and trained to use PlusCare at our 2 participating study sites: 11 CMs from BCH and 9 CMs from BMC (Table 1). CM demographics are presented in Table 1. Reported professional roles of individuals performing CM duties included CMs (n=2), medical CMs (n=3), nurse practitioners (n=4), clinical social workers (n=3), physicians (n=2), nurses (n=2), pharmacists (n=1), and health services advocate (n=1). CMs owned either an iPhone (17/20, 85%) or Android (3/20, 15%) smartphone, and 15% (3/20) reported spending >7 hours on their mobile phones per day. On a scale from 1 to 10 for tech savviness, CMs reported an average score of 7.1 (SD 1.2) for their own tech savviness compared with 6.4 (SD 1.9) for a rating of perceived tech savviness in their workplace. An average score of 6.7 (SD 2) was reported for workplace effectiveness using information technology.

A total of 45 AYA living with HIV participants were enrolled across the 2 study sites. Patient demographics at the 2 sites are shown in Table 2. Of the 45 patients, only 8 (18%) were Hispanic and Latino; 34 (76%) were non-Hispanic, and roughly half (23/45, 51%) identified as female. The average age of the AYA living with HIV participant was 20 (SD 2.7) years with education level ranging from 4% (2/20) of individuals with no schooling completed to 9% (4/20) of individuals holding a bachelor’s degree. AYA living with HIV owned an iPhone (40/45, 89%) or Android smartphone (5/45, 11%), with 40% (18/45) of participants reporting >7 hours of phone use per day. Of the 45 AYA living with HIV participants, only 55% (11/20) reported using health/HIV apps. An average score of 8 (SD 1.6) was reported for self-rated tech savviness. Of the baseline demographics, only education was found to be statistically different between patients at the 2 sites, with a higher proportion of patients with a high school education or less at BMC (68% vs 35%; *P*=.04).

**Table 1:** Case manager (N=20) demographics by site—Boston Medical Center (BMC) and Boston Children’s Hospital (BCH).

Demographic			BCH (n=11)		BMC (n=9)		Total		*P* value	
**Age (** **years), n (%)**										.39
	18-24	3 (27)		1 (11)		4 (20)				
	25-34	4 (36)		1 (11)		5 (25)				
	35-44	0 (0)		2 (22)		2 (10)				
	45-54	1 (9)		2 (22)		3 (15)				
	≥55	3 (27)		3 (33)		6 (30)				
**Ethnicity and race, n (%)**										.82
	Hispanic and Latino	1 (9)		0 (0)		1 (5)				
	Non-Hispanic White	6 (55)		6 (67)		12 (60)				
	Non-Hispanic Black	3 (27)		1 (11)		4 (20)				
	Non-Hispanic other (including American Indian or Alaska Native, Asian and Pacific Islander, or more than one)	0 (0)		1 (11)		1 (5)				
	Unknown	1 (9)		1 (11)		2 (10)				
**Sex, n (%)**										.19
	Female	11 (100)		7 (78)		18 (90)				
	Male	0 (0)		2 (22)		2 (10)				
**Education—highest level, n (%)**										.39
	Bachelor’s degree	5 (45)		2 (22)		7 (35)				
	Master’s degree	2 (18)		5 (56)		7 (35)				
	Doctoral degree	3 (27)		1 (11)		4 (20)				
	Professional degree	1 (9)		1 (11)		2 (10)				
**Smartphone ownership, n (%)**										>.99
	iPhone	9 (82)		8 (89)		17 (85)				
	Android	2 (18)		1 (11)		3 (15)				
**Hours spent on phone per day, n (%)**										.11
	0-3	4 (36)		5 (56)		9 (45)				
	4-6	2 (18)		6 (67)		8 (40)				
	>7	3 (27)		0 (0)		3 (15)				
**Tech savvy, mean (SD)**										.88
	Personal score	7.09 (1.45)		7 (1)		7.05 (1.23)				
	Work score	6 (2.05)		6.89 (1.54)		6.4 (1.85)				
Workplace IT use, mean (SD)			6.36 (2.11)		7.11 (1.9)		6.7 (2)		.30	
**Number of patients**										.42
	Mean (SD)	37.44 (17.7)		43.7 (24.9)		38.95 (22.4)				
	Median (range)	49 (10-54)		43.5 (2-80)		43.5 (2-80)				

**Table 2:** Patient demographics by site—Boston Medical Center (BMC) and Boston Children’s Hospital (BCH; N=45).

Patients		BCH (n=20)	BMC (n=25)	Total	*P* value	
**Age (years)**						.84
	Mean (SD)	20.21 (2.5)	20.04 (2.9)	20.11 (2.7)		
	Median (range)	20 (16-26)	20 (14-25)	20 (14-26)		
**Ethnicity and race, n (%)**						.32
	Hispanic and Latino	2 (10)	6 (24)	8 (18)		
	Non-Hispanic White	3 (15)	1 (4)	4 (9)		
	Non-Hispanic Black	9 (45)	15 (60)	24 (53)		
	Non-Hispanic other (including American Indian or Alaska Native, Asian and Pacific Islander, or more than one)	4 (20)	2 (8)	6 (13)		
	Unknown	2 (10)	1 (4)	3 (7)		
**Sex, n (%)**						.64
	Female	11 (55)	12 (48)	23 (51)		
	Male	9 (45)	13 (52)	22 (49)		
**Education—highest level, n (%)**						.04
	High school graduate or less	7 (35)	17 (68)	24 (53)		
	Some college credit or more	12 (60)	8 (32)	20 (44)		
	N/A^a^ (missing)	1 (5)	0 (0)	1 (2)		
**Income (US $), n (%)**						.13
	<20,000	5 (25)	13 (52)	18 (40)		
	20,000-49,999	6 (30)	7 (28)	13 (29)		
	≥50,000	5 (25)	2 (8)	7 (16)		
	N/A (missing)	4 (20)	3 (12)	7 (16)		
**Time in years since positive HIV diagnosis, n (%)**						.91
	1-3	4 (20)	6 (24)	10 (22)		
	3-5	1 (5)	5 (20)	6 (13)		
	>5	15 (75)	14 (56)	29 (64)		
**Smartphone ownership, n (%)**						.06
	iPhone	20 (100)	20 (80)	40 (89)		
	Android	0 (0)	5 (20)	5 (11)		
**Hours spent on phone per day, n (%)**						.46
	0-3	4 (20)	4 (16)	8 (18)		
	4-6	10 (50)	9 (36)	19 (42)		
	>7	6 (30)	12 (48)	18 (40)		
**Health or HIV apps, n (%)**						.73
	Yes	4 (20)	7 (28)	11 (24)		
	No	15 (75)	17 (68)	32 (71)		
	N/A (missing)	1 (5)	1 (4)	2 (4)		
**Tech savvy**						.15
	Personal score, mean (SD)	8.3 (1.63)	7.6 (1.50)	7.9 (1.58)		
	N/A (missing), n (%)	0 (0)	1 (4)	1 (2)		

^a^N/A: not applicable.

### Use, Usability, and Acceptability

#### Overview

Average SUS scores for CMs and patients were 51 (SD 7.9; n=13) and 63 (SD 10.6; n=38), respectively. We found intersite differences in overall use as measured by the number of forms submitted through the app; of the 290 forms submitted by CMs and 427 forms submitted by patients at both sites, the majority were submitted by BMC participants (624/717, 87% and 667/717, 93%, respectively). CMs used the asynchronous messaging feature with patients most frequently (197/290, 67.9%), followed by updating patient information (53/290, 18.3%), and uploading documents (32/290, 11%). CMs also used the app to upload laboratory results (6/290, 2.1%), add patient notes (1/290, 0.3%), and access the how-to guide (1/290, 0.3%). Among the 427 forms submitted, patients most commonly sent messages to CMs (228/427, 53.4%) and viewed and shared documents (149/427, 34.9%). Patients also updated their personal information (30/427, 7%) and viewed their laboratory results or laboratory results graphs (15/427, 3.5%); they rarely accessed resources (4/427, 0.9%) and the how-to guide (1/427, 0.2%).

**Figure 2:**
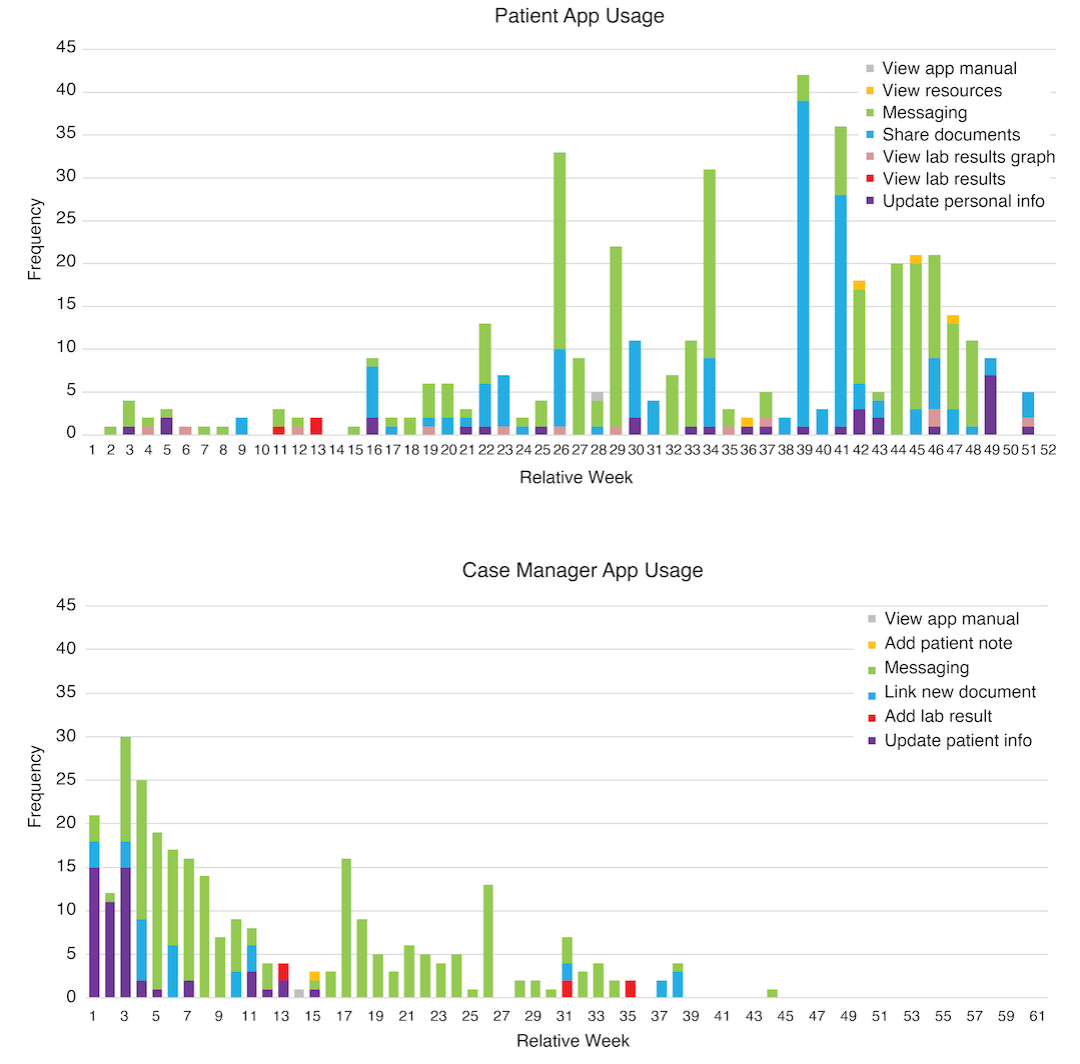
Frequency of patient and case manager app use by feature over week from participant enrollment date (top and bottom, respectively).

Use data revealed that CM use was highest at the beginning of the intervention period, primarily owing to the enrollment of patients and increased use of messaging starting in week 3; however, use dropped to near 0 approximately after 9 of the 15 months. Patient use data revealed a different pattern—a slow increase in use early in the trial period with spikes of activity in the middle to later weeks, with messaging and document management being the most frequent tasks (Figure 2).

Content analyses of the CM interviews at the end of the study revealed potential facilitators, barriers, and recommendations for improving the usability of the app.

#### Facilitators, Barriers, and Recommendations for Use

Overall, CMs found the app helpful in communicating with patients. Several CMs perceived PlusCare as an alternative way to reach patients and send appointment reminders. At one site, CMs used PlusCare to send positive affirmations to patients via SMS text messages sent through the system. CMs reported that patients seemed to appreciate these messages and remarked that support for bidirectional messaging was particularly helpful for them, consistent with the increase in patients using the messaging feature observed in the latter half of the study after the feature was implemented. Many CMs also reported having the ability to share documents and laboratory results as useful features of PlusCare, although use data did not show frequent sharing of results.

A frequently cited barrier to using PlusCare was the inability of patients to respond or initiate messages. Although 2-way, asynchronous messaging was implemented partway through the study in August in response to the need for this capability, use data showed no remarkable increase in the CM use of the system after implementation. In addition, 22% (4/18) of CMs reported that without a read receipt, it was frustrating to know if a patient received a message. A couple described the experience as sending a message out to a “black hole” or into “oblivion.” Consequently, some CMs switched to using alternative methods of contacting patients, such as calling them.

A total of 39% (7/18) of CMs reported that PlusCare was not easy to use or generated extra work. For example, a few CMs found that using the app through the web link (including logging in and uploading documents) was cumbersome and having to manually synchronize the app (a feature inherent in the CommCare platform upon which it is built to enable offline data collection) before each use was a burden. Some CMs also disliked the way notification badges were implemented and indicated that it would have been preferable for the badge activity to reflect the individual’s own activity, as opposed to the activity of the CMs as a group.

Other reasons for low to no CM app use included that the app was not relevant to the tasks they performed with the patients and that their patients did not use the app. Some CMs did not have easy access to a computer or tablet with the app installed or linked to and would have preferred a true mobile phone app connection rather than a web link used on the study tablet. Another reason was the preference for other methods of communication (ie, in person or over the phone) because these methods are perceived as more personal and responses are immediate.

Despite these barriers, many CMs remarked that PlusCare has potential and made recommendations for improvement. To address some of the aforementioned barriers, several CMs suggested adding read receipts to messages, updating the messaging feature to facilitate “real-time” or synchronous conversations, and adding the option to send messages to an individual CM or a designated group of CMs. Other recommendations included support for telehealth visits, more support for platform-agnostic features, and interoperability with other systems, such as electronic medical records. A CM felt that the app was a “great idea,” but its implementation could be further improved by considering additional patient and provider inputs.

### Health and Clinical Outcomes

We assessed the changes in viral load, CD4 count, and clinic visits between the preintervention and postintervention periods. There was no significant change in viral load (N=45; mean 32, 95% CI 17.9-60.4 vs mean 24.7, 95% CI 12.1-47.9; *P=*.30) or CD4 count (N=45; mean 996, 95% CI 857-1157 vs mean 1049, 95% CI 898-1224; *P=*.18) over time. Although there was an 8% absolute increase in the median appointment adherence rate, defined as actual completed appointments of the total number of expected appointments (completed appointments, no shows, and cancelations) in the postintervention period compared with the preintervention period, this change was not significant (*P=*.13).

Mean self-reported monthly medication adherence ratings across 12 months were 4.7 (SD 0.23) out of 6. The fitted regression model was medication adherence rating of 4.8+0.01x, and the overall regression was not found to be statistically significant (*R*^2^=0.1; *F*_1,11_=1.24; *P*=.29). Although in general, good adherence was reported, qualitative feedback from CMs nonetheless suggested that medication adherence remains one of the biggest problems with the AYA living with HIV they worked with and about one-third of AYA living with HIV at one site reported “medication” or “reminder” when sharing potential outcomes or improvements that could be achieved with better use of technology for HIV management, suggesting there remains potential value for mHealth systems to support this aspect of care.

**Figure 3:**
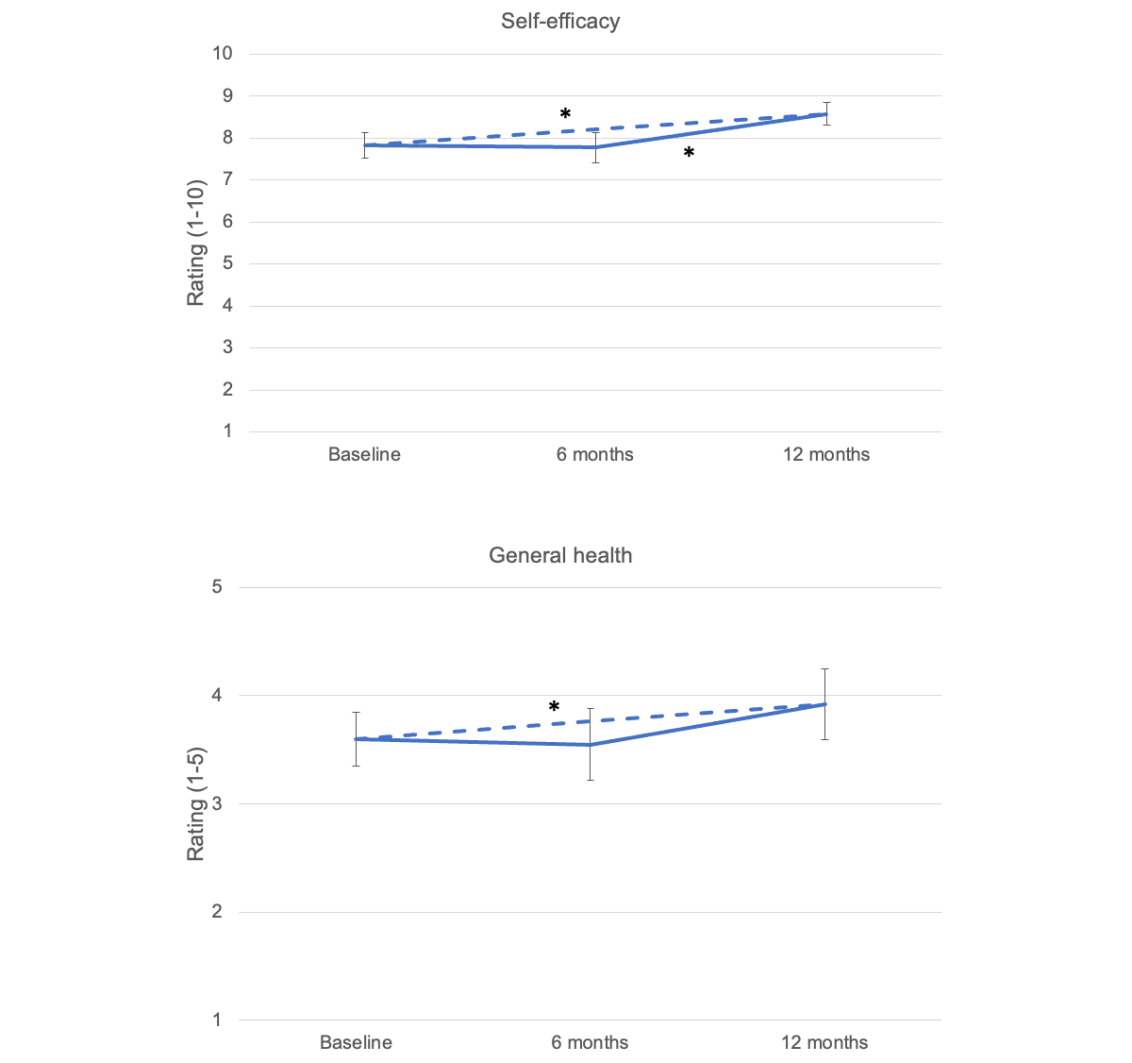
Average (95% CIs) ratings of self-efficacy in chronic disease management (1=not at all confident to 10=totally confident) and health-related quality of life (1=poor to 5=excellent) among youth living with HIV (top and bottom, respectively). Dashed line represents change between baseline and 12-month time points, solid lines represent comparisons between baseline and 6-month and 6- and 12-month time points. Significant increases in self-efficacy were found from baseline to 12 months (*P*=.02) and from 6 to 12 months (*P*=.02). Self-rated general health at 12 months was significantly higher than that at baseline (*P*=.03). Significant increases in ratings between time points are indicated (*).

Patient self-efficacy in chronic disease management and quality of life were assessed at 3 time points over the duration of the study trial (number of patient responses are indicated in parentheses): baseline (n=45), 6 months (n=40), and 12 months (n=38). We found a significant increase in self-efficacy from baseline to 12 months (mean 0.61, SD 0.28; *P=*.02) and from 6 to 12 months (mean 0.65, SD 0.20; *P=*.02) but not from baseline to 6 months (Figure 3). Qualitative feedback from several CMs suggested that PlusCare can help AYA living with HIV take more ownership over their health. CMs at BMC noted that sending affirming messages to patients they felt struggled with medication adherence and self-esteem seemed to have a positive effect on patients’ perceived self-worth and self-esteem.

Self-rated general health at 12 months was significantly higher than that at baseline (mean 0.32, SD 0.14; *P=*.03). However, no significant differences were found from baseline to 6 months and from 6 to 12 months (Figure 3).

We evaluated cost savings by comparing the total per-patient clinic charges during the 12-month pre-enrollment and postenrollment period at each of the 2 sites for emergency department, outpatient, and inpatient visits. Among these charges, the emergency department charges at both sites did not show any decrease between the preintervention and postintervention periods. However, results for inpatient and outpatient charges were mixed. A significant decrease of 65% in inpatient charges was found at BCH (*P=*.03) and a slightly significant decrease of 41% was found in total charges (including emergency, inpatient, and outpatient charges; *P*=.046); however, no decrease was found in outpatient charges alone. No decrease in inpatient charges and small, but not significant (*P*=.24), decrease of 18% in outpatient and 9.5% in total charges (*P*=.37) were found at BMC.

## Discussion

### Principal Findings

In summary, we developed a digital app, PlusCare, to support HIV case management for AYA living with HIV and tested its use with CMs and AYA living with HIV patients at 2 private, not-for-profit academic hospitals. The app was designed to facilitate sharing and viewing of laboratory results, sending and signing documents, updating contacts, scheduling and sending messages, and viewing community resources. We found that both CMs and AYA living with HIV used the messaging feature most frequently and qualitative feedback from CMs suggested that direct messaging between CMs and AYA living with HIV can be particularly useful. There were no significant changes in HIV viral suppression (ie, viral load and CD4 count), appointment, or medication adherence over the course of the study. Slight increases in AYA living with HIV self-reported self-efficacy in chronic disease management and quality of life were observed. Results of the cost-saving analyses were mixed and varied by site.

In part owing to the ubiquity of smartphones among adolescents in the United States, mHealth holds great potential in helping AYA living with HIV overcome barriers to receiving care. mHealth interventions for adults have been studied and shown to have a positive effect on retention and clinical outcomes [47]. However, few mHealth tools have been developed to directly serve AYA living with HIV who face even greater challenges while transitioning to adult care [8,9]. Less than 15% of systematic reviews have focused on mHealth tools, specifically on support for AYA living with HIV [47-49]. Today’s community-based case management approach has become increasingly interdisciplinary, whereby a team of health professionals may play a role in the collaborative process of working with a given client throughout the HIV care continuum [12,50-52]. Recognizing the need for patient and care team coordination, we developed, tested, and deployed a shared platform for clinical care team members to perform case management duties and the AYA living with HIV they serve.

In this study, higher self-efficacy in chronic disease management and self-reported quality of health were found during the intervention period compared with 1 year prior in a within-participant comparison; however, we found no significant improvements in primary clinical outcomes of interest, that is, viral load or CD4 counts. This may be because our study included mostly healthy patients who were virally suppressed, which limited our ability to detect changes in primary clinical outcomes. No changes in medication adherence were observed, as self-rated medication adherence was moderately high throughout the intervention period and likely affected by other structural barriers not addressed by the app. Trends in the data suggested that PlusCare may be associated with an increase in clinic visit adherence and a decrease in per-patient charges, but neither was found to be significant.

Perhaps most surprisingly, we found lower average perceived usability ratings of the app—particularly for the CM interface—than the industry-average SUS score. There are several possible explanations for these findings. Although the SUS score distribution has been used for benchmarking a vast array of new technologies and more recently shown to be suitable for benchmarking mHealth or digital health apps (excluding apps just for self-monitoring physical activity), it is possible that the benchmark score could be subject to reporting bias in the literature, that is, the file drawer problem, where researchers may be more likely to report only higher usability [34,53]. Low mHealth usability has been found in previous studies in vulnerable, underserved populations based on other usability scales and metrics [54,55]; however, to the best of our knowledge, this is the first time SUS scores have been reported for a digital health app with 2 separate interfaces for 2 types of end users, CMs and AYA living with HIV participants. The usability ratings collected here were also found to be lower than the ratings collected in previous controlled, moderated usability testing conducted on an earlier prototype [30]. Qualitative feedback suggested that the features validated in usability testing may have been subject to limitations realized only when tested in naturalistic, unmoderated settings. As mentioned, a limitation in the handling of messaging between CMs and their patients was addressed earlier in the intervention period, which may have influenced the inverse use patterns, in which CMs used the app more during the early weeks of deployment and patients used the app in the latter half of their participation. We observed a steep decline in CM app use around 3 months after initial use, which is in line with data showing >60% decrease in the number of users 3 months after downloading an app [56]. By contrast, patients were less engaged with the app when they were first enrolled in the study, presumably owing to the inability to send or respond to messages, but they continued to use the app, namely accessing documents and messages, for several months toward the middle and end of their study participation, even when CMs were no longer using the app. This suggested that patients may have found continued utility in the app, but qualitative feedback from CMs suggests that they may have preferred to use other means to communicate with and respond to patients, such as phone calls. CMs reported a strong desire for synchronous 2-way messaging (vs asynchronous) or at least a way for them to be notified if a patient received a message. Improving the utility for CMs could increase the potential for sustained use of PlusCare. There remains need for features that can effectively streamline and simplify CM and AYA living with HIV tasks and better support timely communication when needed.

Another example of enhancing provider-patient communication is the sharing of laboratory results, which even when passively shared, may be best conveyed with an opportunity for immediate follow-up and discussion between provider and patient. CMs suggested that the system was particularly useful in communicating with, including sending positive affirmations to, patients and supporting remote document sharing and sign-off, thereby reducing the need for in-person visits, which has been particularly challenging for patients with longer commutes. This is supported by use data, revealing that messaging and managing documents were the most used features. CM feedback also suggested that the system may be particularly helpful for engaging less-adherent individuals who can benefit from more frequent, directed communication and check-ins supported by a shared platform where multiple CMs can follow-up with a given patient. CMs at one site (BMC) reported that they use a tag team approach where a different CM is scheduled for different time windows to be the point person to follow-up with a nonadherent patient.

The discrepancy in perceived usability and actual use highlights the need to continually assess the acceptability of mHealth tools in real-world settings and to use an iterative design process that incorporates and focuses on system improvements based on direct feedback from users to the extent possible. Opportunities remain for the development of technologies that are responsive to the needs of AYA living with HIV, both in the United States and globally [57-59].

### Limitations

This study was designed to explore the naturalistic use of this system in various settings. As such, participants were encouraged to use the system as needed; however, there were no study requirements to ensure baseline use. Generally, low but variable levels of PlusCare app use were observed at both sites, making it difficult to attribute changes in health and clinical outcomes directly to the use of PlusCare.

Another limitation was the limited qualitative feedback and incomplete survey data from patients compared between the time points. The response rates to 6- and 12-month survey assessments were 89% and 84%, respectively. Qualitative check-ins and end-of-study interviews were conducted only with CMs to probe their experience using the system with their patients, while separate usability feedback was collected from patients via survey. It is possible that direct interviews with patients may have provided additional richer insights into, for example, the observation of inverse trends in use between patients and CMs.

Another limitation was related to the sample of participants in the study. Patients had to have been receiving care for at least a year before enrollment in the study. As such, our study population may have been more committed to managing their HIV care than other AYA living with HIV and thus were generally virally suppressed. Future studies could examine mHealth use across the HIV care continuum by including those who are newly enrolled, as well as those already enrolled in care. In addition, only patients with consistent access to the internet and mobile devices were included, excluding those who could potentially derive the greatest benefit from access to PlusCare.

External and temporal factors, such as changes in case management policies and the increasing age of the patient cohort, may have introduced bias to our results, given the quasi-experimental pre-post study design; thus, we cannot attribute our results solely to the intervention, and it is possible that these other factors may have contributed to any differences reported in the pre-post comparisons. Although it is possible that continuous access to PlusCare over the 12-month period could account for the overall high adherence rates and improvements in general health outcomes, it is difficult to attribute this result to PlusCare without a true comparison to usual care.

### Conclusions

Following user-centered design principles, we designed and tested an HIV case management digital app with CMs and AYA living with HIV and found that access to this system correlated with increased self-efficacy in chronic disease management and health-related quality of life. PlusCare was designed to provide remote support for various day-to-day case management functions and decrease the need for in-person visits. The results suggested trends of decreased patient costs; however, there was no significant decrease in clinic visits or outpatient costs. Although it is difficult to attribute our results directly to the use of the PlusCare app given its low use and the lack of a control group, results from this study suggest that digital health tools supporting case management between the CM and AYA living with HIV hold promise in improving patient quality of life and self-efficacy in chronic disease management—a critical goal of care for CMs working with individuals who will one day need to take full ownership of their own health needs. As health care facilities continue to seek ways to reduce the paperwork burden, a shared digital platform, such as the one designed and studied here for AYA living with HIV and the frontline health workers they work directly with, has the potential to streamline and improve AYA living with HIV care across the United States and globally, in areas with increasing connectivity and access to mobile technology among youth and young adults.

## Appendix

Multimedia Appendix 1 Case manager check-in form.

Multimedia Appendix 2 Case manager end-of-study interview probes.

## Abbreviations

AYA: adolescents and young adults
BCH: Boston Children’s Hospital
BMC: Boston Medical Center
CM: case manager
HRQOL-4: Health-Related Quality of Life-4
IRB: institutional review board
mHealth: mobile health
RA: research assistant
SUS: System Usability Scale
