# Supplementary material for: A Digital Platform to Support HIV Case Management for Youth and Young Adults: Mixed Methods Feasibility Study
Source: JMIR Form Res. 2022 Nov 21;6(11):e39357. doi: 10.2196/39357 (PMC9723976; doi:10.2196/39357)
Supplement: Multimedia Appendix 1 [file formative_v6i11e39357_app1.pdf]

CM 6-Week Check-In Question Form (available for RA to document feedback collected at 6-week timepoint or whenever prompted by CMs)

Instructions to RA: Please use this form to collect feedback from case managers to identify and troubleshoot barriers to usage as needed.

1. CM: [study ID]

2. Please select which of the following activities CM is experiencing difficulties with:
    a. Logging in
    b. Updating patient information
    c. Sending patient lab results
    d. Sharing documents
    e. Sending patient reminders
    f. Updating patient contacts
    g. Composing a patient note
    h. Other

3. [If Other] Other, please describe: _______________

4. Please describe how problem was addressed/resolved: ________________
