# Supplementary material for: A Digital Platform to Support HIV Case Management for Youth and Young Adults: Mixed Methods Feasibility Study
Source: JMIR Form Res. 2022 Nov 21;6(11):e39357. doi: 10.2196/39357 (PMC9723976; doi:10.2196/39357)
Supplement: Multimedia Appendix 2 [file formative_v6i11e39357_app2.pdf]

CM Focus Group/Interview Probes

a. What features did you like the *most* about PlusCare?

b. What features did you like the *least* about PlusCare?

c. What do you feel were some of the greatest benefits of using PlusCare in your practice?

d. What do you feel were some of greatest limitations of using PlusCare in your practice?

e. How does day-to-day care of your patients with PlusCare compare to case day-to-day care without the app?

f. How do you think PlusCare has affected communication between you and your patients?

g. How do you feel PlusCare has affected communication between members of the care team?

h. If you had a choice, would you continue to use PlusCare as it is currently designed?

i. What recommendations do you have for improvement?
